# Four clinical phenotypes of cow’s milk protein allergy based on dairy product specific IgE antibody types in North China

**DOI:** 10.3389/fimmu.2022.949629

**Published:** 2022-10-06

**Authors:** Rui Tang, Xiaohong Lyu, Yi Liu, Mingzhi Zhu, Xukai Yang, Zhoujie Wu, Bingnan Han, Shandong Wu, Jinlyu Sun

**Affiliations:** ^1^ Allergy Department, State Key Laboratory of Complex Severe and Rare Diseases, Peking Union Medical College Hospital, Chinese Academy of Medical Sciences and Peking Union Medical College, Beijing, China; ^2^ Allergy Department, Beijing Key Laboratory of Precision Medicine for Diagnosis and Treatment of Allergic Diseases, National Clinical Research Center for Dermatologic and Immunologic Diseases, Peking Union Medical College Hospital, Chinese Academy of Medical Sciences and Peking Union Medical College, Beijing, China; ^3^ Eight-year program of clinical medicine, Chinese Academy of Medical Sciences, Peking Union Medical College, Beijing, China; ^4^ Hangzhou Zheda Dixun Biological Gene Engineering Co., Ltd., Hangzhou, China; ^5^ Zheda Dixun Anti-Allergy Functional Molecular Laboratory, Department of Development Technology of Marine Resources, College of Life Sciences and Medicine, Zhejiang Sci-Tech University, Hangzhou, China

**Keywords:** cow’s milk allergy, IgE, Fermented milk, boiled milk, raw milk

## Abstract

**Background:**

Cow’s milk protein allergy (CMPA) is a common allergy. Immunoglobulin E (IgE)-mediated cow’s milk allergy is associated with a high mortality risk and poor prognosis. The study aims to investigate whether there are different clinically CMPA phenotypes in China and to explore the association between CMPA phenotypes and specific IgE (sIgE) antibodies against different dairy products.

**Methods:**

Serum sIgE against different animal milk and cow’s milk products and different milk components was measured by an allergen array. Four CMPA classifications were identified by the presence of serum sIgE: boiled milk-positive, yogurt-positive, buttermilk-positive, and raw milk-positive.

**Results:**

Among the 234 participants included in the study, 9 were boiled milk sIgE-positive, 50 were yogurt sIgE-positive, 17 were buttermilk sIgE-positive, and 158 were only raw milk sIgE-positive. The boiled milk-positive group had the highest levels of raw milk sIgE and casein sIgE antibodies, followed sequentially by the yogurt-positive, buttermilk-positive, and raw milk-positive groups. The boiled milk group observed the highest levels of sIgE against raw milk, casein, α-lactalbumin, and β-lactoglobulin. These levels differed significantly from those in the other three groups. Allergic symptoms were distributed differently among the four study groups. The percentages of allergic patients with gastrointestinal tract symptoms in the above mentioned four groups ranged from high to low, and the percentages of patients with skin symptoms in the four groups ranged from low to high, respectively.

**Conclusion:**

Based on dairy product sIgE antibody levels associated with different milk components and various clinical allergic symptom tendencies, we could distinguish four CMPA phenotypes.

## Introduction

Food allergies are a health problem of increasing concern worldwide. Food allergy is defined as a specific immune response caused by a portion of specific food, which can occur repeatedly and cause disorders involving the skin or gastrointestinal or respiratory tract. Milk, eggs, wheat, soybeans, peanuts, nuts, fish, and shellfish are considered to be the common causes of food allergy (1). Cow’s milk protein allergy (CMPA) is a specific immune response to various proteins in milk. CMPA can be attributed to immunoglobulin E (IgE)-mediated and non-IgE-mediated mechanisms (2–8). CMPA is the most common food protein allergy in infants and children (9). In previous studies, the prevalence of IgE-mediated CMPA was 2%-9% (9). A prospective study of 6768 children with suspected CMPA conducted jointly by seven hospitals in southern China showed that 182 children were diagnosed with CMPA, and the prevalence of CMPA was 2.69% (10).

A food allergy may endanger life safety and greatly affect quality of life. An accurate food allergy diagnosis is necessary to avoid severe allergic reactions and unnecessary dietary restrictions. However, accurate diagnosis can be difficult. According to practice guidelines (11), the initial diagnosis of CMPA should be based on the exclusion of milk protein from the diet. Then, a double-blind placebo-controlled oral food challenge (OFC), the gold standard for the diagnosis of CMPA (3, 12), should be performed. However, the trial requires the cooperation of patients and parents, which is time-consuming and expensive. It is challenging to implement under real-world conditions in China. Even nondouble-blind OFC has the risk of an immediate or severe allergic reaction (13).

There are diagnostic methods for diagnosing CMPA other than OFC, including screening scoring systems, skin prick tests (SPTs) and serum milk-specific Immunoglobulin E (sIgE) detection. However, the cutoff point value of the milk-related symptom score has not been agreed upon in the academic community, and the sensitivity and specificity of this diagnostic tool are low (14). The area under the receiver operating characteristic (ROC) curve (AUC) was only 0.68 (14). An SPT is used to detect the presence of IgE tissue-binding antibodies, which can reflect IgE-mediated allergic diseases to a certain extent. However, this test is prone to false-negative results in infants because infants usually have a poor response to the SPT (15). Because of the convenience and ability to detect various allergens, serum sIgE detection (16–20) is widely used to assist in the diagnosis of CMPA in China.

CMPA patients need to strictly avoid milk in daily life to prevent the occurrence of an allergic reaction. However, this common management practice has coincided with the delayed resolution of the allergy. Strict milk avoidance does not improve long-term outcomes and significantly affects patients’ quality of life. Many CMPA patients believe from experience that they can tolerate some milk-containing products. Kim JS et al. reported that ingesting extensively heated milk products (baked milk) accelerates and increases the overall likelihood of milk allergy resolution in CMPA patients without significant adverse effects (21). Furthermore, Cansin Sackesen et al. found that CMPA patients had different reactivity levels to baked, fermented, and whole milk according to a new Luminex-based peptide assay (22). It seems that CMPA patients could be divided into different phenotypes according to reactivity to different milk products (23).

We designed this cross-sectional study to investigate whether there are different clinically CMPA phenotypes in North China and to explore the association between the CMPA phenotypes and sIgE antibodies against different dairy products. In addition, we investigated the clinical characteristics of the different CMPA phenotypes.

## Materials and methods

### Study design and patients

This study was a cross-sectional study. Participants with CMPA underwent evaluation at the department of allergy at Peking Union Medical College Hospital between May 2019 and July 2019. Clinical information was collected through either a questionnaire or chart review. Written informed consent was obtained. CMPA was documented when a patient reported a convincing history of acute reaction after food ingestion in the previous 12 months, considering the results of milk sIgE, SPT, or a previous positive OFC result. The study was approved by the Institutional Ethics Committee of Peking Union Medical College Hospital (JS-3427).

### Allergy evaluation

Detection of serum sIgE against different animal milks, different cow’s milk products and different milk components was performed with an allergen array (Hangzhou Zheda Dixun Biological Genetic Engineering Co., Ltd.). The lower and upper detection limits were 0.0 and 1000 kU/L, respectively. Serum sIgE values of 0.35 kU/L (4) or higher were considered indicative of positive results. The animal milks included raw milk, buffalo’s milk, goat’s milk, and mare’s milk. The cow’s milk products included boiled milk (representative of extensively heated milk), hydrolyzed milk powder, yogurt, cheddar cheese, buttermilk, defatted milk powder, cottage cheese, and cow whey. The cow’s milk components included casein, α-lactalbumin, β-lactoglobulin and bovine serum albumin (4).

The items of serological testing used in this study include allergenic component proteins of milk-casein (NA-BD8-1), alpha-lactalbumin (NA-BD4-1), beta-lactoglobulin (NA-BD5-1) and bovine serum albumin (NA-BD6-1) purified through HPLC or colum chromatography were purchased from Indoor biotechnologies. On the other hand, food allergen of raw milk, buffalo’s milk, goats’ milk, mare’s milk, boiled milk, hydrolyzed milk powder, yogurt, cheddar cheese, buttermilk, defatted milk powder, cottage cheese and cow whey are extract from natural food.

For protein extraction from natural foods, 10 mL of raw milk/buffalo’s milk/sheep’s milk/mare’s milk/boiled milk was centrifuged at 4000 rpm and 4°C for 15 min, add 50 mL of cold acetone to degrease, remove the acetone and air dry, add 100 mL of Coca’s solution (0.1M Na2CO3/NaHCO3, 0.1M NaCl, 2mM EDTA, 20mM DIECA) to the air-dried sample and stir overnight. After centrifugation at 10,000 rpm and 4°C for 30 min, the supernatant was taken for ultrafiltration. The ultra-filtered liquid was centrifuged at 12,000 rpm and 4°C for 30 min, and the supernatant was collected. Then the dialysis was carried out in PBS at 4°C under magnetic stirring for 10 h to obtain the final milk extract.

10 g of hydrolyzed milk powder/yogurt/cheddar cheese/buttermilk/defatted milk powder/cottage cheese/cow whey powder was dissolved in 100 mL purified water. 10 mL was taken for extraction. The extraction method was the same as raw milk.

Three hundred microlitre of undiluted serum is applied on chips wetted by washing buffer in the kit and incubated for 45 min, the secondary antibody is applied and incubated for 45 min after washing five times, then enzyme solution is applied and incubated for 20 min after washing five times. All steps are operated at room temperature. The DX-allergen Analysis Software (version 7.0) export testing results and interpretation of the results was based on international classification standards (24). The allergen specific IgE antibody contents are calculated according to a typical standard curve established with WHO IgE human serum (3rd International Standard) 11/234 (25).

The methodology is based on antigen-antibody specific reaction. The antigen proteins are immobilized on the matrix membrane. During the experiment, the antibody in the serum specifically binds to the corresponding antigen on the matrix membrane. After removing excess serum, a secondary antibody that recognizes human antibodies is added and then washed. An enzyme that can be linked to the secondary antibody is added, and this enzyme can undergo a chromogenic enzymatic reaction with the substrate. Determine the concentration of allergen-specific antibodies in serum based on the degree of colour development.

The comparative results of the sIgE test kit in this study and ImmunoCAP-250 system (Thermo Fisher Scientific) were a positive coincidence rate of 100.00% and a negative coincidence rate of 84.62%. The sIgE test kit in this study was also used in previous studies which have been published (26, 27). Similar sIgE test kit developed by another company was used in published studies also (28, 29).

### Allergic symptom evaluation

According to guidelines recently, there is no symptom that is specific for CMA as its every manifestation can be caused by multiple conditions (30). The participants’ allergic symptoms were evaluated according to five systems. The allergic symptom of the upper respirational tract included allergic rhinitis. Allergic symptoms of the lower respiratory tract included cough and allergic asthma. Allergic symptoms of the skin included urticaria, allergic purpura, rash and blister. Allergic symptoms of the gastrointestinal tract included abdominal pain, diarrhea, and bloody stool caused by food hypersensitivity. Allergic symptoms of severe anaphylactic reactions were severe, life-threatening systemic allergic reactions.

### Statistical analyses

All data were analyzed using SPSS statistical software, version 26.0 for Mac (SPSS Inc, Chicago, IL) and GraphPad Prism version 9.0.0 for Mac (GraphPad Software, San Diego, California USA). Milk sIgE levels were analyzed by ANOVA and t tests. P values of less than 0.05 were regarded as significant.

## Results

### Participants’ characteristics

Two hundred and thirty-four CMPA participants were enrolled. The baseline characteristics of the participants are presented in **Table 1**. The average age of the CMPA participants was 5.8 ± 9.4 years old, ranging from 6 month to 61 years old. There were 151 males and 83 females. Almost all the participants completed all the milk-related sIgE detection examinations, except 49 participants with missing mare’s milk sIgE data and 185 participants with missing defatted milk powder, cottage cheese or cow whey sIgE data. More details regarding the numbers of milk-related product sIgE-positive participants are provided in **Table 1**.

**Table 1:** Demographic characteristics.

Participant number, n	234
Male: Female	151:83
Age (average, range)	6, 0-61
**Number participants positive for sIgE against different animal milks**	
Raw milk -n (%)	234/234 (100.0%)
Buffalo’s milk -n (%)	65/234 (27.8%)
Goats’ milk -n (%)	62/234 (26.5%)
Mare’s milk -n (%)	2/185 (1.1%)
**Number of participants positive for sIgE against different cow’s milk products**	
Boiled milk -n (%)	9/234 (3.9%)
Hydrolyzed milk powder -n (%)	29/234 (12.4%)
Yogurt -n (%)	59/234 (25.2%)
Cheddar cheese -n (%)	48/234 (20.5%)
Buttermilk -n (%)	43/234 (18.4%)
Defatted milk powder -n (%)	35/49 (71.4%)
Cottage Cheese -n (%)	17/49 (34.7%)
Cow whey -n (%)	33/49 (67.4%)
**Number of participants positive for sIgE against different cow’s milk components**	
Casein -n (%)	23/234 (9.8%)
α-lactalbumin-n (%)	24/234 (10.3%)
β-lactoglobulin-n (%)	106/234 (45.3%)
bovine serum albumin-n (%)	119/234 (50.9%)

### Serum sIgE antibodies against raw milk and other milk-related products

**Figure 1:**
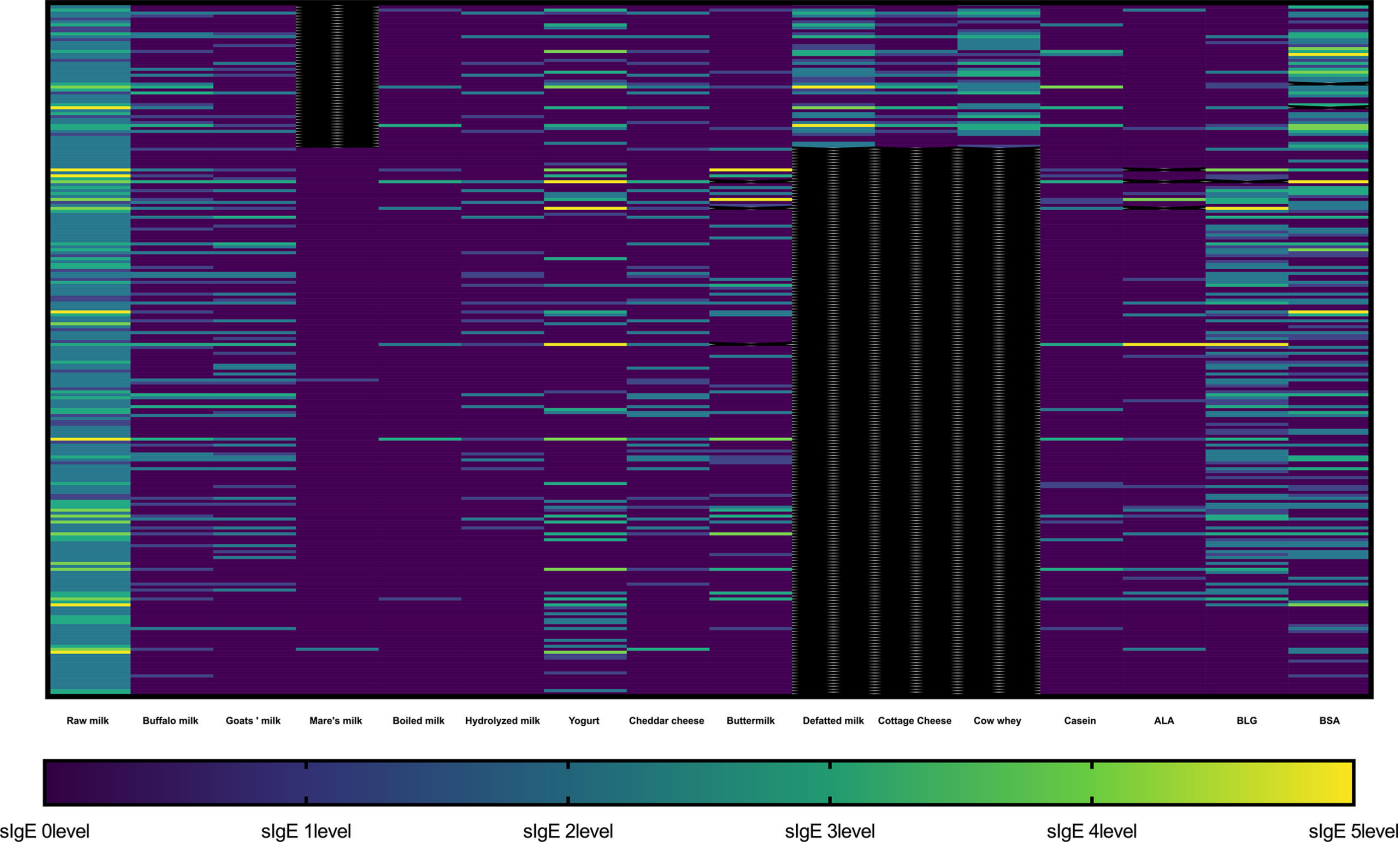
Heatmap representing sIgE levels against different milk-related product in each participant. The sIgE result was divided into 6 levels: level 0 (< 0.35 kU/L), level 1 (≥ 0.35 kU/L and < 0.7 kU/L), level 2 (≥ 0.7 kU/L and < 3.5 kU/L), level 3 (≥ 3.5 kU/L and < 17.5 kU/L), level 4 (≥ 17.5 kU/L and < 50 kU/L), level 5 (≥ 50 kU/L and < 100 kU/L), and level 6 (≥ 100 kU/L). The black color represents missing data. ALA, α-lactalbumin; BLG, β-lactoglobulin (BLG); BSA, bovine serum albumin (BSA).

A heatmap of the sIgE results of the participants is shown in **Figure 1**. The heatmap indicated that the positive results of sIgE against various dairy products were consistent with that of raw milk.

### Exploration of CMPA phenotypes

**Figure 2:**
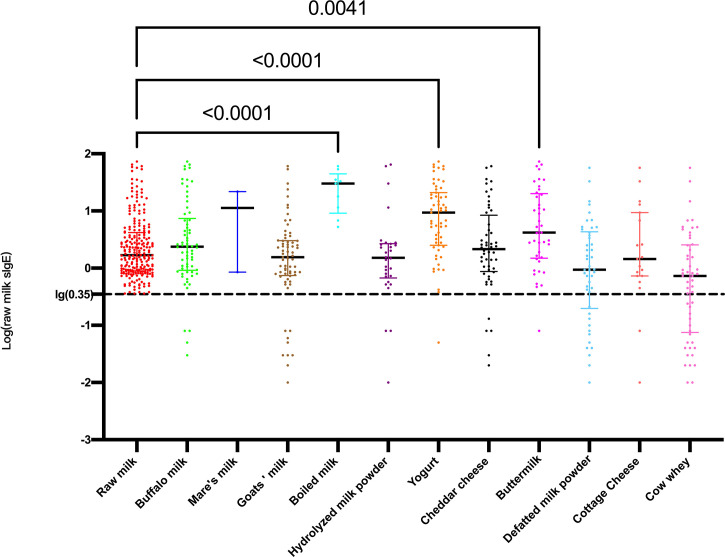
Levels of log-transformed sIgE against raw milk among different dairy product sIgE-positive participants.

The comparison of the raw milk sIgE levels among the raw milk sIgE-positive participants and the other dairy sIgE-positive participants is shown in **Figure 2**. The raw milk sIgE level in boiled milk sIgE-positive participants was highest, with a median of 30.13 and interquartile range (IQR) of 9.165 to 44.550, which was significantly higher than that in the raw milk sIgE-positive participants (median 1.685, IQR 0.898-4.183, *P*<0.0001). In addition, the raw milk sIgE levels in the yogurt- and buttermilk-positive participants were significantly higher than those in the raw milk-positive participants (*P*<0.0001 and *P*=0.0041). Therefore, reactivity to cow’s milk in participants in the boiled milk-positive group, yogurt-positive group, buttermilk-positive group, and raw milk-positive group were likely different. It is reasonable to explore the detailed milk component sIgE results and the clinical characteristics of these four groups.

**Figure 3:**
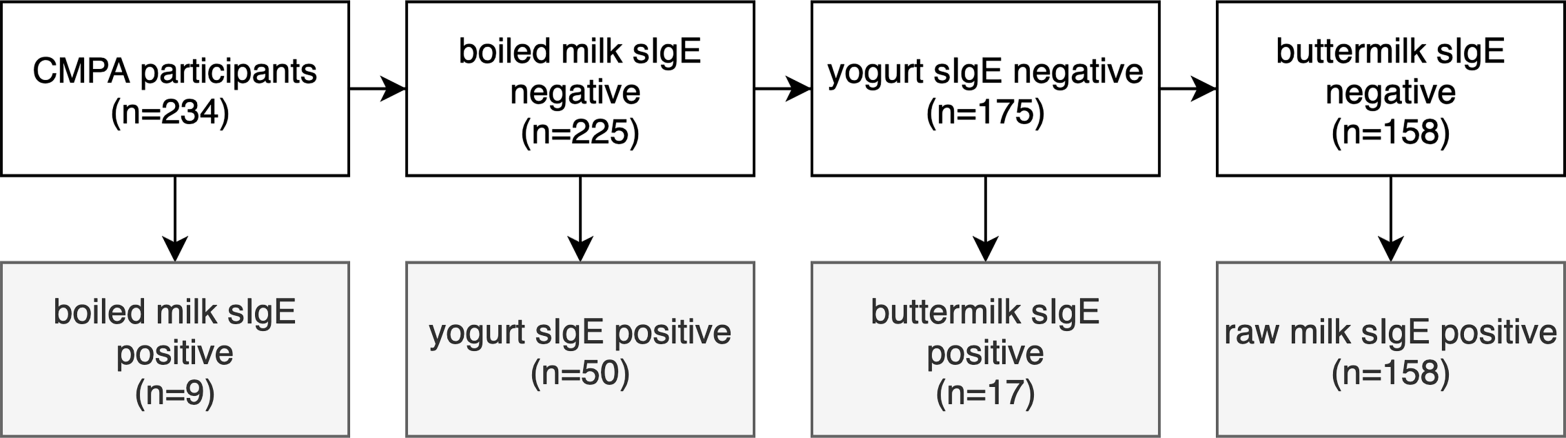
Flow diagram showing the distributions of participants according to the boiled milk, yogurt, butter milk and raw milk sIgE results.

Among the 234 participants included in the study (**Figure 3**), 9 were boiled milk sIgE-positive. Fifty were boiled milk sIgE-negative but yogurt sIgE-positive. Seventeen were boiled milk and yogurt sIgE-negative but buttermilk-sIgE positive. One hundred fifty-eight were boiled milk, yogurt, and buttermilk sIgE-negative but raw milk sIgE-positive. These four groups were the main study groups of this study.

### sIgE antibodies against raw milk and milk components in the study groups

**Figure 4:**
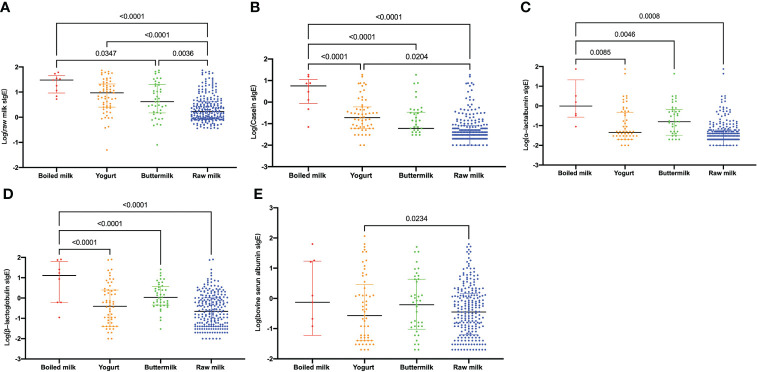
Levels of log-transformed sIgE among the four groups, namely, the boiled milk, yogurt, buttermilk, raw milk. **(A)**; casein **(B)**; α-lactalbumin **(C)**; β-lactoglobulin **(D)**; bovine serum albumin **(E)**. The graph shows medians and quartiles.

The levels of antibodies against raw milk and casein were significantly different across the four clinical milk allergy groups (boiled milk, yogurt, buttermilk and raw milk) (**Figure 4A, B**). The highest levels were observed in the boiled milk group, and these levels differed significantly from those in the other three groups; this difference was more pronounced and significant for the levels of sIgE against raw milk (**Figure 4A**), casein (**Figure 4B**), α-lactalbumin (**Figure 4C**) and β-lactoglobulin (**Figure 4D**). In contrast, the raw milk group observed the lowest levels for raw milk, casein, α-lactalbumin, and β-lactoglobulin. The casein sIgE level in the yogurt and buttermilk groups were lower than those in the boiled milk group (*P*<0.0001, **Figure 4B**).

### Allergic symptoms in the four study groups

**Figure 5:**
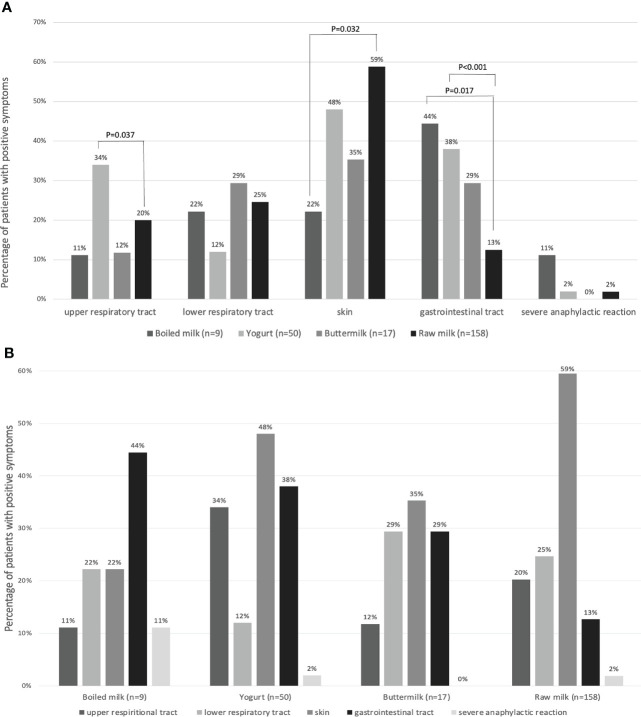
**(A)** The percentages of patients with allergic symptoms in four groups: boiled milk (n = 9), yogurt (n = 50), buttermilk (n = 17), and raw milk (n = 158), reported as groups by affected body systems. **(B)** The percentages of patients with allergic symptoms, reported as four groups.

As shown in **Figure 5**, allergic symptoms were distributed differently among the four study groups. The percentage of participants with upper respiratory tract allergic symptoms in the raw milk group was 20% (32/158), which was significantly lower than that in the yogurt group (34%, 17/50) (*P*=0.037). The percentage of participants with skin allergic reactions in the raw milk group was highest (59%, 94/158), which was significantly higher than that in the boiled milk group (22%, 2/9, *P*=0.032). The percentage of participants with gastrointestinal tract allergic symptom in the boiled milk group was highest (44%, 4/9), significantly higher than that in the raw milk group (*P*=0.017). In addition, there was also a higher percentage of patients with gastrointestinal tract symptoms in the yogurt group than in the raw milk group (*P*<0.001). There was a higher percentage of severe anaphylactic reactions in the boiled milk group than in the other groups, although this did not reach statistical significance.

## Discussion

In this cross-sectional study, we sought to differentiate distinct clinical phenotypes of CMPA according to traditional serum dairy product sIgE levels. The identified dairy product sIgE sequence (boiled milk, yogurt and buttermilk, raw milk) differentiated four classifications of participants:

- Patients who reacted to boiled milk;
- Patients who were negative for boiled milk sIgE and positive for yogurt sIgE;
- Patients who were negative for boiled milk sIgE, negative for yogurt sIgE, and positive for buttermilk sIgE;
- Patients who were negative for boiled milk sIgE, negative for yogurt sIgE, negative for buttermilk sIgE, and positive for raw milk sIgE.

Moreover, as previously reported, the raw milk sIgE levels and casein sIgE levels among the four groups were significantly different from high to low. Allergic symptoms were distributed differently among the four groups.

In this study, we proposed a classification of CMPA based on dairy product sIgE levels in North China for the first time. Boiled milk is an extensively heated milk product usually referred to as baked milk in other studies (22, 31). Extensively heated milk-tolerant subjects with negative extensively heated milk sIgE results have been shown to have lower levels of IgE against casein proteins, lower IgE-binding diversity to milk epitopes (32–37), and a better prognosis of CMPA resolution. A recent study reported the occurrence of intermediate-severity milk product allergy based on reactivity to fermented milk products such as yogurt and cheese. Therefore, the field gradually realizes the classification of clinical CMPA. However, there have been no studies on CMPA phenotypes in China. This classification based on sIgE results may not only help divide CMPA patients into allergic risk groups for management but also help increase quality of life, for example, by reducing stress levels and promoting social communication with others (38).

Several clinical trials have investigated heated milk tolerance in children with milk allergy (39, 40). Nowak-Wegrzyn et al. (31) conducted a study in children aged 2-17 years with CMPA. The children were challenged with extensively heated milk products. Seventy-five percent of the children with CMPA could tolerate the extensively heated milk product but not unheated milk. The 25% who reacted to the extensively heated milk product had significantly larger SPT wheals and higher milk-specific and casein-specific IgE levels than those in the other groups. In this study, as shown in **Figure 4**, in the boiled milk group, the levels of sIgE against raw milk, casein, α-lactalbumin, and β-lactoglobulin were significantly higher than those in the other three groups. In addition, the milk component sIgE levels in the yogurt- or buttermilk- positive patients were higher than those in the only raw milk sIgE-positive patients but lower than those in the boiled milk sIgE-positive patients. The mechanism might be related to the thermal processing of cow’s milk (41). Thermal processing can disrupt conformational IgE-binding epitopes but not usually continuous IgE-binding epitopes, which seems to play a role in milk allergy. In addition to altering IgE epitopes, thermal processing may change different biophysical and immunological properties of food proteins, such as their structure, function, solubility and digestibility and the T cell response.

This study is the first to report the allergic symptoms in CMPA patients among these four groups differentiated by sIgE levels. Wipa Jessadapakorn et al. reported that among patients with cow’s milk allergy, the casein sIgE level in the urticaria group tended to be higher than that in the atopic dermatitis (AD) group (42). Arianna Giannetti et al. reported that patients with AD showed higher rates of polysensitization to foods and higher levels of both total IgE and sIgE against milk, casein, wheat, peanuts, and cat dander at different ages than patients without AD (43). Although the classification of clinical CMPA was explored in a previous study, the levels of sIgE against different dairy products in allergic patients with different symptoms were evaluated. However, the distribution of clinical allergic symptoms was not reported according to the new classification. As seen in **Figure 5**, the percentages of allergic patients with gastrointestinal tract allergic symptoms among the following four groups were as follows, from high to low: boiled milk group, yogurt group, buttermilk group, and raw milk group. Interestingly, the percentages of patients with allergic skin reactions followed the opposite sequence. The boiled milk sIgE-positive patients had the lowest percentage of skin symptoms but the highest percentage of gastrointestinal tract symptoms. This order was the opposite in only raw milk sIgE-positive patients. We performed pairwise comparisons between each pair of groups, and more details are shown in **Figure 5**.

The method to detect the sIgE of different dairy product is an allergen array. The cutoff threshold was >=0.35 kU/L in this study. Although several studies might argue about the cutoff in sIgE test (44), which reported that the level of IgE positivity using 0.35 represented sensitization rather than actual allergy. However, the diagnosis of the patients with CMPA in this study was based on a combination of clinical presentation, sIgE, past medical history, SPT, or previous OFC results. According to previous guideline and studies, it was reasonable to adopt the traditional cutoff 0.35 (3, 4). In addition, in recent studies, there was also some other new method to identify milk allergens also. Fierro’s study measured the traces of milk and/or egg allergens in biscuits by two different liquid-chromatography-mass spectrometry methods (45).

There are some limitations to this study. All the diagnoses of CMPA were based on medical histories, clinical symptoms, sIgE levels and previous diagnoses instead of an OFC. It is difficult to carry out OFCs in China because of immature technology, patient resistance and the danger associated with the test. In addition, the sample was limited to the boiled milk sIgE-positive group, although the total sample was relatively large. All of the intergroup comparisons were performed by strict statistical tests to reduce the limitation of sample size. The low boiled milk sIgE positivity rate might suggest the rarity of this CMPA subtype. Another limitation of this study was that the lack of CMPA onset age or evolution years of these patients, which might imply clinical manifestations.

In conclusion, the new dairy product sIgE sequence (boiled milk, yogurt or buttermilk, raw milk) differentiated four groups of CMPA participants. Among the CMPA patients in the four groups, the levels of sIgE against raw milk and casein tended to range from high to low, according to the sequence above. The percentages of allergic patients with gastrointestinal tract symptoms in the four groups also ranged from high to low. However, the percentages of patients with skin symptoms in the four groups ranged from low to high. This study helps remind clinicians to pay attention to boiled milk, yogurt, and buttermilk sIgE-positive CMPA patients. It might be reasonable to adopt stratified measures for the management of patients who are positive for different dairy product, for example, stricter for boiled milk allergy patients.

## Data availability statement

The original contributions presented in the study are included in the article/Supplementary Material. Further inquiries can be directed to the corresponding authors.

## Ethics statement

The studies involving human participants were reviewed and approved by Institutional Ethics Committee of Peking Union Medical College Hospital. Written informed consent to participate in this study was provided by the participants’ legal guardian/next of kin. 

## Author contributions

JS, BH, and SW designed and directed the project. YL, MZ, XY, ZW performed the experiments. XL analyzed data. RT and XL wrote the article. All authors contributed to the article and approved the submitted version.

## Funding

Research Application of Egg and Milk Allergen Component Diagnostic Series Products (Hangzhou science external specialties 2021 No. 79).

## Conflict of interest

Authors YL, MZ, XY, ZW, SW are employed by Hangzhou Zheda Dixun Biological Gene Engineering Co., Ltd.

The remaining authors declare that the research was conducted in the absence of any commercial or financial relationships that could be construed as a potential conflict of interest.

## Publisher’s note

All claims expressed in this article are solely those of the authors and do not necessarily represent those of their affiliated organizations, or those of the publisher, the editors and the reviewers. Any product that may be evaluated in this article, or claim that may be made by its manufacturer, is not guaranteed or endorsed by the publisher.
